# The P2X7 Receptor Stimulates IL-6 Release from Pancreatic Stellate Cells and Tocilizumab Prevents Activation of STAT3 in Pancreatic Cancer Cells

**DOI:** 10.3390/cells10081928

**Published:** 2021-07-29

**Authors:** Lara Magni, Rayhana Bouazzi, Hugo Heredero Olmedilla, Patricia S. S. Petersen, Marco Tozzi, Ivana Novak

**Affiliations:** Section for Cell Biology and Physiology, Department of Biology, University of Copenhagen, 2100 Copenhagen, Denmark; lara.magni@bio.ku.dk (L.M.); rayhana.bouazzi@bio.ku.dk (R.B.); hugo.heredero.olmedilla@gmail.com (H.H.O.); patricia@sund.ku.dk (P.S.S.P.); marco.tozzi@sund.ku.dk (M.T.)

**Keywords:** pancreatic cancer, PDAC, pancreatic stellate cells, IL-6, Tocilizumab, P2X7R, STAT3, fibrosis, eATP

## Abstract

Pancreatic stellate cells (PSCs) are important pancreatic fibrogenic cells that interact with pancreatic cancer cells to promote the progression of pancreatic ductal adenocarcinoma (PDAC). In the tumor microenvironment (TME), several factors such as cytokines and nucleotides contribute to this interplay. Our aim was to investigate whether there is an interaction between IL-6 and nucleotide signaling, in particular, that mediated by the ATP-sensing P2X7 receptor (P2X7R). Using human cell lines of PSCs and cancer cells, as well as primary PSCs from mice, we show that ATP is released from both PSCs and cancer cells in response to mechanical and metabolic cues that may occur in the TME, and thus activate the P2X7R. Functional studies using P2X7R agonists and inhibitors show that the receptor is involved in PSC proliferation, collagen secretion and IL-6 secretion and it promotes cancer cell migration in a human PSC-cancer cell co-culture. Moreover, conditioned media from P2X7R-stimulated PSCs activated the JAK/STAT3 signaling pathway in cancer cells. The monoclonal antibody inhibiting the IL-6 receptor, Tocilizumab, inhibited this signaling. In conclusion, we show an important mechanism between PSC-cancer cell interaction involving ATP and IL-6, activating P2X7 and IL-6 receptors, respectively, both potential therapeutic targets in PDAC.

## 1. Introduction

Pancreatic ductal adenocarcinoma (PDAC) is one of the most aggressive and lethal cancers and it is increasing in incidence [1,2]. The 5-year relative survival rate is lower than 10%, depending on tumor stage at the time of diagnosis. The poor prognosis is due to the lack of symptoms in early stages, and consequent late diagnosis, as well as the limited effect of present therapies and metastatic spread. PDAC presents itself as a solid tumor with a complex tumor microenvironment (TME) that contributes to immunosuppression and poor efficiency of chemo- and radiotherapy [3]. The TME is made up of cellular and non-cellular components, including cancer-associated fibroblasts, cancer infiltrating immune cells, extracellular matrix, various cytokines, and nucleotides, which all can interact with cancer cells and support tumor growth, immunosuppression, and metastasis [4,5].

Pancreatic stellate cells (PSCs) are the main fibroblast-like cell type present in the TME. In the healthy pancreas, PSCs are quiescent and show vitamin-A containing lipid droplets [6]. Upon transient activation, as in response to injury, inflammation and other types of stress, PSCs are responsible for tissue repair. However, with continuous activation, they contribute to pathogenic processes, such as carcinogenesis [7]. In PDAC, there is a two-way crosstalk between PSCs and pancreatic cancer cells [8,9]. Firstly, cancer cells activate PSCs by paracrine stimulation, mediated by platelet-derived growth factor (PDGF), fibroblast growth factor (FGF2), and transforming growth factor (TGF-β1) [10], leading to an increase in PSC migration, proliferation and collagen release [11]. Secondly, once activated, PSCs, in turn, release growth factors and cytokines that support cancer growth and cancer cell migration [12]. For example, PSCs release a cytokine, Interleukin 6 (IL-6), which has been proposed as a key factor in the PSC-PDAC interplay [13,14]. IL-6 binds to IL-6 receptors (IL-6R) to promote signal transduction involving the Janus kinase/signal transducers and activators of the transcription 3 (JAK-STAT3) pathway in a number of cells [15]. Recently, it was shown that IL-6 secreted by human and murine PSCs stimulated STAT3-dependent cancer cell survival, migration, and metastasis [13,14,16]. In addition, serum levels of IL-6 are higher in mouse models of pancreatic cancer (KPC) and PDAC patients [17]. However, which factors elevate IL-6 levels?

Some of the other components present in the microenvironment of several tumors are extracellular nucleotides/-sides such as pro-inflammatory extracellular ATP (eATP) and immunosuppressive adenosine [4,5,18,19]. This is most likely also the case for PDAC [4,20,21], and since a number of pancreatic cells (cancer cells, PSCs, and immune cells) express various P2 and adenosine receptors, extensive purinergic signaling can be found within the TME [4]. One of the relevant purinergic receptors is the P2X7 receptor (P2X7R), as it is involved in several patho-/physiological processes, such as pancreatic cancer [22,23], inflammation [24] and pain [25]. The P2X7R belongs to the P2X ion channel/receptor family and is encoded by a highly polymorphic gene [26,27,28,29] with some single nucleotide polymorphisms correlating with human diseases (chronic lymphocytic leukemia, osteoporosis and pain) [27,30,31]. Moreover, there are also different splice isoforms of the P2X7R, A-J in humans and A and K in rodents, where A are regarded as the common full-length variants [26,32]. The P2X7R is activated by eATP at relatively high concentrations (mM), leading to the formation of a cytolytic pore [5]. However, lower concentrations of ATP (μM) can also activate the P2X7R and have in turn shown positive effects on, e.g., cell proliferation and migration [22,23]. The underlying mechanism(s) in these seemingly opposing effects of the P2X7R remains unsettled [24,33]. Our previous studies have implicated the P2X7R as an important factor in the pathophysiology of PDAC. The P2X7R is expressed in human pancreatic cancer cells and mouse PSCs and shows this dual effect in in vitro models [22,34,35]. In an orthotropic PDAC cell model, targeting of the P2X7R decreased tumor fibrosis and tumor activity, highlighting the importance of purinergic signaling/P2X7R interplay in the TME in vivo [23]. Most interestingly, co-culture of mouse PSCs with human cancer cells indicated that PSCs released a yet unidentified chemoattractant in a P2X7R-dependent manner [23].

Our hypothesis is that purinergic–cytokine signaling, in particular P2X7R–IL-6–STAT3 signaling, could be important in the interplay between PSCs and cancer cells in the TME of PDAC models. The aim of the present study was first to show whether the P2X7R is expressed and functional in human PSCs and compare it to the receptor in murine PSCs. The second aim was to elucidate whether IL-6, the well-established cytokine in PDAC tumor progression, could be our chemoattractant candidate released by PSCs and stimulating pancreatic cancer cells. For this purpose, we used a human PSC (hPSC) line, RLT-PSC, and primary murine PSCs (mPSCs) isolated from mice with either wild type P2X7R (P2X7R ^wt^) or loss-of-function P2X7R with a proline to leucine mutation in the C-terminal domain of the receptor (P2X7R ^P451L^). This study shows that activation of the P2X7R in PSCs leads to IL-6 release into the TME and subsequent activation of STAT3 signaling in pancreatic cancer cells. Potentially, inhibition of the P2X7 receptor may present a possible therapeutic strategy to target both pancreatic cancer and stellate cells by preventing their crosstalk and tumor progression.

## 2. Materials and Methods

### 2.1. Cell Culture and Chemicals

For the purpose of this study, we used both primary mouse PSCs (mPSCs) and a human pancreatic stellate cell line, RLT-PSC (hPSC) [36]. The mPSCs were taken from male mice with either wild type P2X7R (P2X7R ^wt^) or loss-of-function P2X7R with a proline to leucine mutation at amino acid 451 (P2X7R ^P451L^) on BALB/c and C57BL/6 backgrounds, respectively (Taconic Biosciences, Ejby, DK). Isolation of mPSCs was carried out as described previously [34]. All experiments were performed on euthanized animals, which were handled in accordance with the EU directive 2010/63/EU on protection of animals used for scientific purposes and experimental protocols and were approved by the Danish Animal Experiments Inspectorate (license no. 2011/561–56). The human pancreatic ductal adenocarcinoma cell line (PANC-1) was purchased from American Type Culture Collection (ATCC, Manassas, VA, USA, CRL-1469). Cells were grown in standard high glucose (25 mM) Dulbecco’s modified Eagle’s medium (DMEM GlutaMAX supplement, Thermo Fisher Scientific, Tåstrup, DK, 31966-047) supplemented with 10% fetal bovine serum (FBS) and incubated at 37 °C with 5% CO_2_. P2X7R function was studied using the specific allosteric inhibitor AZ10606120 (Tocris, Bristol, UK, 3332/10), the competitive antagonist A438079 (Tocris, 2972) and the agonist 2′(3′)-O-(4-Benzoylbenzoyl) adenosine 5′-triphosphate triethylammonium salt, BzATP (Sigma, Søborg, DK, 112898-15-4).

### 2.2. Western Blots

Protein lysate was prepared from mouse and human PSCs for P2X7R detection. Samples were boiled at 98 °C for 10 min in the presence of 50 mM dithiothreitol (DTT). Proteins for STAT3 activation analysis were obtained using lysis buffer enriched with the protease-phosphatase inhibitor kit (Thermo Fisher Scientific) and boiled for 3 min at 95 °C. Samples were prepared with 50 mM DTT and heated for 5 min at 95 °C. Protein samples (30–40 μg) were loaded on a polyacrylamide gel (NuPAGE 10% Bis-Tris 10-well, Thermo Fisher Scienific), and then transferred to a PVDF membrane (Invitrogen, Thermo Fisher Scienific). Membranes were blocked in TBS-T buffer + 5% skim milk for 1 h at room temperature and incubated overnight at 4 °C with primary antibody against the P2X7R (APR-004, Alomone Labs, Jerusalem, IL, 1:500, RRID: AB_2040068), Vinculin (#V9131, Sigma, 1:1000, RRID: RRID:AB_477629), β-Actin (Sc-47778, Santa Cruz, Tilst, DK, 1:1000, RRID: AB_626632), pSTAT3 (#9145, Cell Signaling, Herlev, DK, 1:1000, RRID: AB_2491009) or STAT3 (#9139, Cell Signaling, 1:1000, RRID: AB_331757). Membranes were then incubated with secondary HRP-conjugated antibodies (1:2000, EZ-ECL-Biological Industries, Fredensborg, DK) and visualized with Fusion FX (Vilber Lourmat, Eberhardzell, DE).

### 2.3. Real Time PCR

mRNA from mouse and human PSCs was isolated with RNeasy Micro Kit (QIAGEN, Copenhagen, DK) following the manufacturer’s instructions. For RT-PCR, 1 μg total RNA was used for one-step RT-PCR (QIAGEN) using primers for P2X7R (Table 1).

Amplification parameters as follows: one cycle at 50 °C for 30 min and one cycle at 95 °C for 15 min followed by 37 cycles at 94 °C for 1 min, 58 °C for 1 min, 72 °C for 1 min, and finally, one cycle at 72 °C for 10 min.

### 2.4. Immunofluorescence

Proteins were visualized using immunofluorescence (IF). Cells were split (20,000 cells/mL for hPSCs and 30,000 cells/mL for mPSCs) to coverslips in a 6-well plate and allowed to attach overnight. Next, cells were fixed with paraformaldehyde (4% in PBS) for 15 min, washed 3 × 5 min with PBS and then treated with 0.1 M TRIS-glycine buffer at pH 7.4 for 15 min. Permeabilization of cells was carried out by washing 3 × 5 min with 0.3% TritonX-100 in PBS and a blocking step of 40 min with PBS + 5% BSA was carried out before the addition of the primary antibody (see Table 2) in PBS + 1% BSA overnight at 4 °C. The following day, the slides were washed 3 × 5 min with PBS + 1% BSA before incubation for 1 h with the secondary antibody (Table 2) in PBS + 1% BSA. The slides were then washed 1 × 5 min with PBS before incubation with DAPI (D3571, Thermo Fisher Scientific, RRID: AB_2307445) in PBS + 1% BSA for 15 min. Lastly, the slides were washed 3 × 5 min with PBS before mounting on Menzel-Gläzer microscope slides with a small drop of Dako fluorescent mounting medium. Fluorescence was examined with 40 × 1.3 NA or 63 × 1.2 NA objectives in Leica TCS SP5 X confocal microscope (Leica Microsystems, Heidelberg, DE). Images were analyzed in Leica software.

### 2.5. Calcium Signals

PSCs were seeded in Wilco dishes and incubated with 5 μM Fura-2 AM (Invitrogen) for 30 min in a physiological buffer without bicarbonate (–BIC) contained in mM: 140 NaCl, 1 MgCl_2_·6H_2_O, 1.5 CaCl_2_·2H_2_O, 0.4 KH_2_PO_4_, 1.6 K_2_HPO_4_·3H_2_O, 5 glucose and 10 HEPES, pH 7.4. Subsequently, cells were washed and equilibrated in a –BIC buffer and experiments were conducted at 37 °C. PSCs were stimulated with BzATP and/or ATP and for control with 1 μM ionomycin or digitonin. Fura-2-loaded cells were illuminated at λex = 340 nm and λex = 380 nm (60 ms, 1 s intervals) using a TILL Polychrome monochromator. Emission was collected at 500 nm by an image-intensifying, charge-coupled device camera (Andor X3 897, Belfast, UK) and digitized by a FEI image processing system (Thermo Fischer Scientific). The intracellular Ca^2+^ transients were depicted as the ratio of Fura-2 fluorescence signals recorded at 340/380 nm.

### 2.6. Cell Proliferation

The proliferation rate of PSC types was first detected using the Cell Counting Kit-8 (CCK-8, Sigma) and later the Cell Proliferation ELISA, BrdU kit (Roche, Hørsholm, DK). Cells (4000) were grown in a 96-well plate at 1% or 0% FBS for 24 h prior to 48 h stimulation with agonist or inhibitor (given in chemicals). Cells were incubated with CCK-8 or with BrdU kit reagents (1.5 h for hPSCs and 4 h for mPSCs) according to the manufacturer’s instructions. Absorbance and chemiluminescence, respectively, were measured in a FLUOstar Optima microplate reader (BMG Labtech, Ortenberg, DE, USA).

### 2.7. Cytotoxicity and Cell Viability Assay

The cytotoxicity assay was performed with the In Vitro Toxicology Assay Kit, Lactate Dehydrogenase based (Sigma) according to the manufacturer’s instructions. hPSCs (4000) were grown in a 96-well plate for 24 h prior to 48 h stimulation with agonist or inhibitor in 0% FBS. Absorbance was measured in a FLUOstar Optima microplate reader. Cell viability was measured using the flow cytometry and the Dead Cell Apoptosis Kit with Annexin V (AV) Alexa Fluor 488 and Propidium Iodide (PI) (Molecular Probes, Invitrogen). hPSCs (50,000–100,000) were plated and allowed to attach for 24 h prior to 48 h stimulation with agonist or inhibitor in 0% FBS-media. After incubation, cells were harvested and stained for 15 min with annexin V Alexa Fluor 488 and PI. The staining was visualized on FlowSight imaging flow cytometer (Merck-Millipore).

### 2.8. Collagen Detection

mPSC (BALB/c) and hPSCs (40,000) were grown in a 24-well plate for 24 h prior to 24 h stimulation with agonist or inhibitor and 5 μM Aphidicolin (Sigma) in 0% FBS media. Collagen was detected with the Sirius Red/Fast Green Collagen Staining kit (Chondrex, Redmond, WA, USA). Absorbance was measured in a FLUOstar Optima microplate reader and the extracellular collagen levels were calculated according to the manufacturer’s instructions.

### 2.9. IL-6 Release

IL-6 released was measured in mPSCs and hPSCs using IL-6 ELISA Novex kit and V-PLEX Human IL-6 Kit (MSD, Copenhagen, DK), respectively. A measure of 20,000 cells were seeded in a 24-well plate. After 24 h, the media was discarded and the cells were treated with agonists and/or inhibitors diluted in 0% FBS media. hPSCs were treated with 5 μM Aphidicolin to induce a cell-cycle arrest. Aliquots of the media were collected after 24 h and IL-6 was measured according to the manufacturer´s instructions.

### 2.10. Cell Migration and Co-Culture

PANC-1 cell migration was monitored using a Boyden-chamber assay. PANC-1 cells (8000) were plated in the upper chamber of the insert (uncoated, transparent PET membrane, 8.0 µm pore size, Falcon, Sigma) with 5 μM Aphidicolin in 1% FBS medium. In the lower chamber, culture medium with 1% FBS was added with agonist or inhibitor. After 24 h, cells were fixed in cold methanol and stained with Crystal Violet. Bright field images were taken with 10× objective in a Leica DMI6000B microscope. Cells were counted using ImageJ (version 1.47, National Institute of Health, Bethesda, MD, USA).

For co-culture of PANC-1 and hPSCs, hPSCs (30,000) were plated in the lower compartment of the Boyden chamber in complete media and let to attach. After 24 h, the media was replaced with 1% FBS media containing 5 μM Aphidicolin and either with agonist or inhibitor. At the same time, PANC-1 cells (8000) were plated in 1% FBS media in the upper chamber of the insert as above. After 24 h, migrated PANC-1 cells were fixed in cold methanol for 10 min, stained with Crystal Violet, visualized and counted as above.

### 2.11. Conditioned Media

hPSCs (1000000) were seeded in complete media in small culture flasks. After 24 h, the medium was replaced with 1% FBS medium supplemented with 5 μM Aphidicolin and agonist or inhibitor. After 24 h, the conditioned media were collected. PANC-1 cells (500000) were plated in a 6-well plate in complete media and allowed to attach. After 24 h, the PANC-1 medium was replaced with the conditioned media from the hPSCs and incubated at 37 °C for 30 min. Proteins were extracted and phosphorylated STAT3 (pSTAT3), total STAT and β-actin were quantified with Western blot.

### 2.12. IL-6R Neutralization with Tocilizumab

The conditioned media collected from hPSCs, as described above, was incubated 1 h at 37 °C with/without 10 ng/mL Tocilizumab (Roche). PANC-1 cells were seeded, and after 24 h, treated with 700 μL of 1% FBS medium ± 10 ng/mL Tocilizumab and incubated for 1 h at 37 °C. Conditioned media were added and cells were incubated for 30 min. Protein extraction was followed by Western blot and pSTAT3/STAT3 quantification.

### 2.13. ATP Release Assay

ATP release was monitored using the ATP kit SL (BioThema, Handen, SE, USA, 11–501). hPSCs (5000) or PANC-1 cells (10,000) were plated in a white 96-well plate with clear plastic bottoms and allowed to attach for 24 h in complete media. After attachment, the cells were washed twice with a physiological –BIC buffer (see above). Cells were allowed to rest in 65 μL of –BIC at 37 °C, then 25 μL of D-luciferin/luciferase enzyme mix was added very carefully to each well and luminescence was measured with FLUOstar Optima luminometer (BMG Labtech, Ortenberg, DE, USA). After reaching a stable baseline for at least 120 s, cells were stimulated mechanically or with a compound and ATP release was monitored for about 10 min. Compounds (glucose, mannitol) were pipetted gently to avoid mechanical disturbances. The addition of a –BIC buffer to the well served as a “mechanical disturbance” control and the value was subtracted from the “stimulated” effect. Mechanical stimulation was performed by injecting 50 μL of –BIC using the FLUOstar’s injection pump (260 μL/s). For each experiment, an ATP standard curve was made using dilution of an ATP stock (BioThema) in –BIC in the range of 0.65 nM to 6.5 μM. The number of cells were determined using CCK-8 assay in parallel wells. To convert the relative luminescence units (RLU) to concentrations of ATP, a power regression curve was fitted to the ATP standard curve and then adjusted to the number of cells in the well. A change in ATP release (ΔATP) was calculated as ATP (M/10^6^ cells/mL) by subtracting the baseline from the peak value after stimulation. For each value, the average of replicates was used.

### 2.14. Statistical Analysis

Non-normalized data were tested with one-way ANOVA with subsequent Bonferroni correction. Normalized data were analyzed with one-sample *t*-tests, followed by correction for multiple comparisons with the Holm–Bonferroni method, when more than two different conditions were tested relative to the control. Comparisons between single treatments and combinations were analyzed with two-tailed unpaired *t*-test.

## 3. Results

### 3.1. Human and Murine PSCs Express the P2X7 Receptor

**Figure 1:**
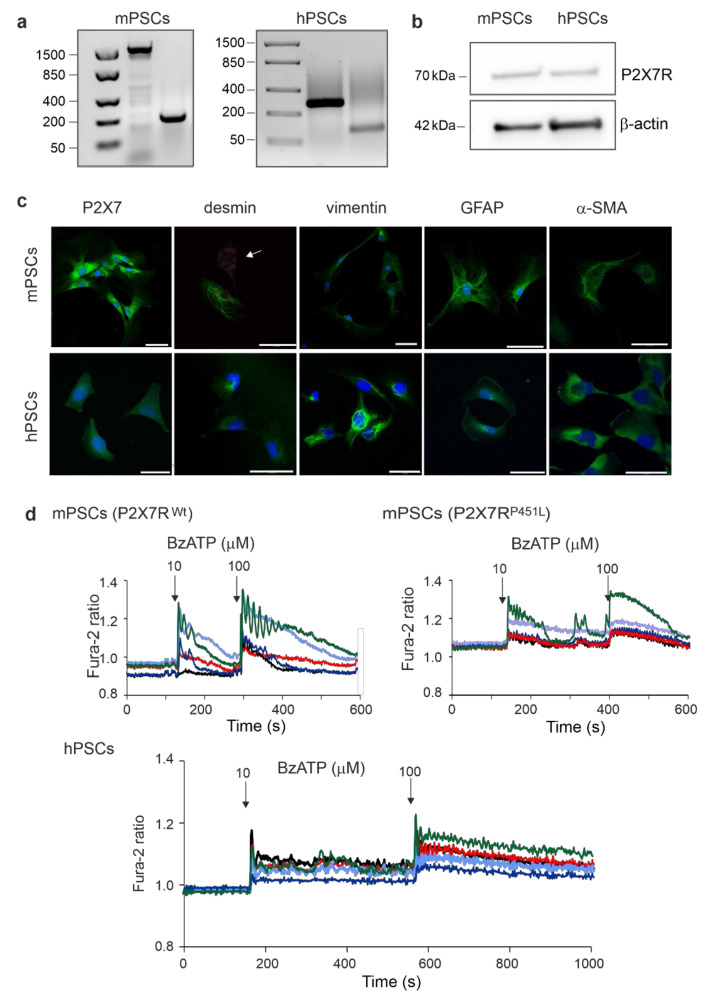
Expression of P2X7R, PSC markers and calcium signals in mPSCs and hPSCs. (**a**) RT-PCR results with different pairs of primers for the P2X7R (Table 1). (**b**) Representative blots of P2X7R (70 kDa) isoform A and the loading control β-actin. (**c**) Immunofluorescence detection of P2X7R and the PSC markers desmin, vimentin, GFAP and α-SMA. Image of mPSCs stained for desmin also shows less activated/quiescent cells with autofluorescent lipid droplets (arrow). Scale bar is 50 μm in all images. (**d**) Fura-2 ratio in PSCs after consecutive stimulation with 10 μM BzATP and 100 μM BzATP. The graph shows 5 representative cells out of 10–20 cells recorded per experiment, 6 experiments.

The first objective was to find whether our model of hPSCs, RLT-PSCs, express the P2X7R. The P2X7R expression was determined on mRNA and protein levels using RT-PCR and Western blot analysis, respectively (Figure 1a,b). RT-PCR results show band sizes for mPSC and hPSC consistent with the primers used (Table 1), while the Western blot analysis highlights a marked band at 70 kDa, which corresponds to the full length P2X7R (isoform A). The P2X7R was also detected with immunofluorescence both in hPSCs and mPSCs (Figure 1c). We also tested for expression of other PSCs markers. Using immunofluorescence, we showed that both human and mouse PSCs express the PSC markers desmin, vimentin, GFAP, and α-SMA (Figure 1c). The short-term activation of the receptor was determined in Ca^2+^ imaging experiments. For this purpose, we used an ATP analogue, BzATP, which activates the P2X7R and is about 10 times more potent than ATP in human and mice [25]. Intracellular Ca^2+^ signals, monitored with Fura-2, were evoked with 10 and 100 μM BzATP in mPSCs, as well as in hPSCs (Figure 1d). In mPSCs, the average change in the Fura-2 response with BzATP was about twice as high in cells derived from the P2X7R ^wt^ compared to P2X7R ^P451L^ mice. 

### 3.2. P2X7R Affects Cell Proliferation and Death in PSCs

**Figure 2:**
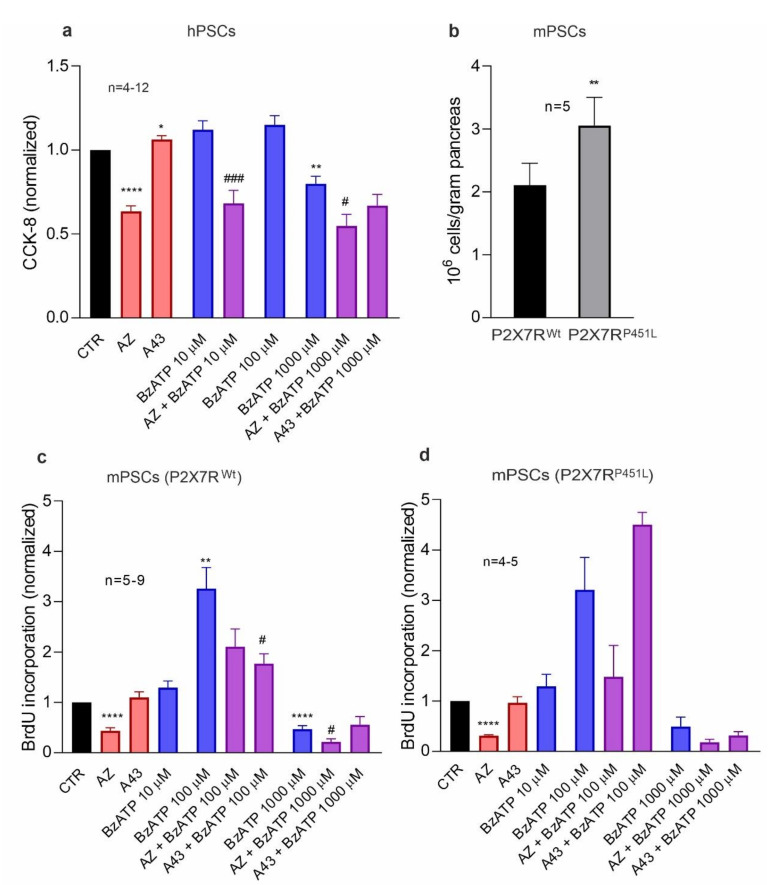
Role of P2X7R in cell proliferation in (**a**) hPSCs and mPSCs from (**c**) P2X7R ^wt^ and (**d**) P2X7R ^P451L^ mice. Cells were treated with different concentrations of the agonist BzATP (0, 10, 100, 1000 μM) with/without 10 μM inhibitor AZ10606120 (AZ) or A438079 (A43) in 1% FBS. Data are normalized to the control value (CTR). We performed one-sample *t*-test followed by Bonferroni correction and significance compared to the control is indicated as follows: * *p* < 0.05, ** *p* < 0.01, **** *p* < 0.0001. Comparisons between agonist and agonist + inhibitor have been evaluated with *t*-test and significance is indicated by # *p* < 0.05, ### *p* < 0.001. (**b**) Number of PSCs isolated from the pancreas of either P2X7R ^wt^ or P2X7R ^P451L^ mice, *p* = 0.0059.

As activated PSCs have an elevated proliferation rate that contribute to tissue repair and under some circumstances to pathogenic processes, we next tested the effect of the P2X7R agonist, BzATP, and the P2X7R inhibitors, the allosteric inhibitor AZ10606120 and the competitive antagonist A438079, on cell proliferation (Figure 2, and Supplementary Materials Figure S1). Since the P2X7R can stimulate basal cell proliferation in HEK293 cells transfected with P2X7R [37], mPSCs [34] and PDAC cells [22] in low serum or serum free medium, we used serum concentrations of 1% and 0% (Figure 2 and Supplementary Materials Figure S1, respectively). When hPSCs were treated with AZ10606120 in basal conditions, there was a significant reduction in cell proliferation (CCK-8 and BrdU assay) in comparison to the control (Figure 2a, Supplementary Materials Figure S1a,b), which indicates that the P2X7R was already activated, similar to our studies on the several pancreatic cancer cell lines [22]. The antagonist A438079 alone did not inhibit proliferation in basal conditions. Treatment with micromolar (10–100 µM) concentrations of BzATP also had no significant effect on cell proliferation, presumably because these cells were already activated in the given culture conditions. However, we observed a significant reduction in cell proliferation with 1000 µM treatment of BzATP, which could be related to the P2X7R pore formation and/or cell death [34,38]. Pre-treatment with A438079 alleviated the inhibitory effect of high-dose BzATP in hPSCs in 0% FBS (Supplementary Materials Figure S1c).

We also evaluated the role of P2X7R in mPSCs obtained from two different mouse strains: P2X7R ^wt^ and P2X7R ^P451L^, with wild type P2X7R or P2X7R with a loss-of-function proline to leucine mutation in the C-terminal, respectively (Figure 2c,d for 1% FBS, Supplementary Materials Figure S1d,e for 0% FBS). Notably, the number of PSCs isolated from the pancreas of P2X7R ^P451L^ mice was about 30% higher than those from P2X7R ^wt^ mice (Figure 2b), indicating that mutation in the receptor increases the PSC number. In agreement with data on hPSC, mPSC proliferation was also significantly reduced with AZ10606120 (Figure 2c,d). This effect was observed in both mice strains. Cell proliferation was increased with BzATP (100 µM) in both cell types. However, at a higher BzATP concentration (1000 µM), cell proliferation decreased below the basal level. The inhibitor, AZ10606120, was able to suppress the proliferative effect of 100 µM BzATP but could not overcome the inhibitory effect of 1000 µM BzATP (Figure 2c–d, Supplementary Materials Figure S1d,e). The inhibitor, A438079, prevented the proliferative effect of 100 µM BzATP in mPSCs from P2X7R ^wt^ mice (Figure 2c, Supplementary Materials Figure S1e), but it was unable to inhibit proliferation in mPSCs from P2X7R ^P451L^ mice. At 1000 µM BzATP, this inhibitor had no further effects.

**Figure 3:**
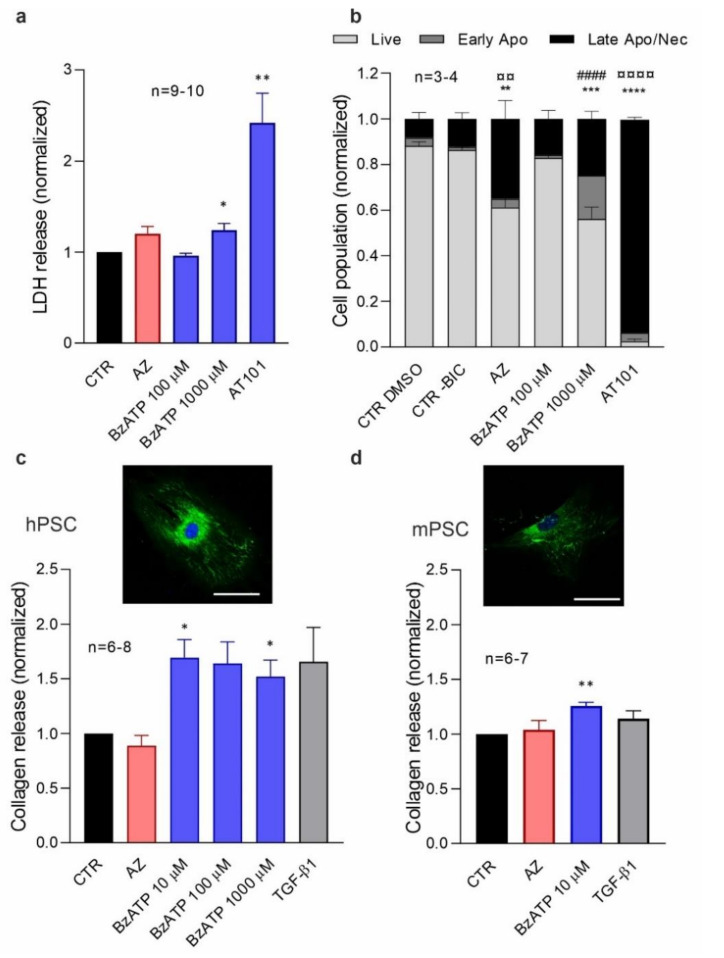
Effect of P2X7R on cell survival and collagen release. (**a**,**b**) Effects of 10 μM AZ10606120 (AZ) and different concentrations of BzATP on (**a**) LDH release (**b**) and live/dead cell population (flow cytometry analysis) on hPSCs. (**c**,**d**) Collagen release and representative images of collagen staining are shown for hPSCs and mPSCs (P2X7R ^wt^). Scale bar is 50 μm. Data are normalized to the control values (CTR). AT101 10 μM and TGF-β1 5 ng/µl were used as positive controls. Statistical analysis was as above and significance is indicated by (**a**) * *p* < 0.05, ** *p* < 0.01; (**c**) * *p* < 0.05; (**d**) ** *p* < 0.01. We analyzed the flow cytometry data with one-way ANOVA followed by Bonferroni correction and compared to the control and significant difference for live (*), early apoptosis (#), late apoptosis/necrosis (¤) is indicated by the following: ** ¤¤ *p* < 0.01; *** *p* < 0.001; **** #### ¤¤¤¤ *p* < 0.0001.

As BrdU incorporation was negatively affected by the P2X7R inhibitor AZ10606120 and BzATP (1000 μM), we wanted to uncover whether this was due to an actual decrease in the proliferation rate or due to cell death; therefore, two different assays were performed on hPSCs: lactate dehydrogenase (LDH) assay and Annexin V/PI stain (Figure 3a,b; Supplementary Materials Figure S2) in order to estimate cellular cytotoxicity and apoptotic/necrotic cell death, respectively. LDH release was slightly but significantly increased after treatment with 1000 μM BzATP (Figure 3a), indicating some cytotoxicity. Flow cytometry analysis showed that this treatment increased a number of cells in early/late apoptosis or necrosis (Figure 3b, Supplementary Materials Figure S2). Therefore, treatment of cells with a millimolar concentration of BzATP caused cell death and alongside decreased cell proliferation in hPSCs, as previously shown in mPSCs [34]. Treatment with AZ10606120 caused a significant shift from live to late-apoptotic/necrotic cell state as detected with Annexin V/PI stain, while the cytotoxic LDH assay only indicated a small (but not significant) increased effect of the treatment (Figure 3b, Supplementary Materials Figure S2). Perhaps arrest in cell proliferation caused cell stress and thus apoptosis/necrosis rather than cytotoxicity. The apoptotic control AT-101 also gave a significant increase in cell death detected in both assays (Figure 3b, Supplementary Material Figure S2).

### 3.3. P2X7R Activation Increases Collagen Release in PSCs

As activated PSCs are known to be the main producers of collagen in areas of fibrosis [7], we wanted to study the role of the P2X7R in this process in hPSCs and mPSCs. Earlier, we showed that the inhibition of the P2X7R with AZ10606120 in an orthotropic PDAC model reduced collagen deposition, presumably through mPSCs [23]. Collagen release was, therefore, quantified both in hPSC and P2X7R ^wt^ mPSC (Figure 3c,d). Stimulation with BzATP (10–1000 μM) significantly increased the collagen release in both hPSC and mPSC, which was comparable to the positive control stimulant TGF-β1. Collagen I was also detected by immunofluorescence (Figure 3c,d inserts). Hence, P2X7R stimulation increased collagen secretion both in hPSC and mPSC.

### 3.4. P2X7R Activation Increases IL-6 Release in PSCs

**Figure 4:**
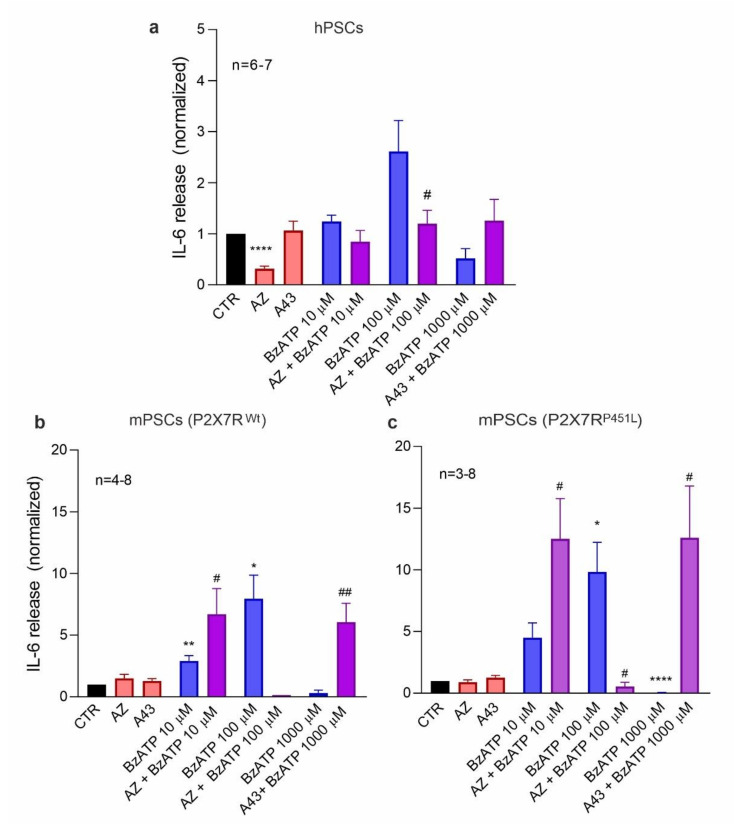
P2X7R -dependent IL-6 release. IL-6 release in (**a**) hPSCs and mPSCs from (**b**) P2X7R ^wt^ and (**c**) P2X7R ^P451L^ mice after treatment with different concentrations of the agonist BzATP (0, 10, 100, 1000 μM) alone and in combination with 10 μM inhibitor AZ10606120 (AZ) and A438079 (A43). Data were normalized to control values (CTR). One-sample *t*-test followed by Bonferroni correction was performed and significance compared to the control is indicated as follows * *p* < 0.05, ** *p* < 0.01, **** *p* < 0.0001.; comparisons between agonist and agonist + inhibitor have been evaluated with *t*-test and significance is indicated by # *p* < 0.05, ## *p* < 0.01. (**b**) n is reported in the graph except for AZ10606120 10 μM + BzATP 100 μM, where n = 2. For hPSCs BzATP 100 μM, uncorrected *p* is 0.045; P2X7R ^wt^ BzATP 1000 μM, uncorrected *p* is 0.057.

PSCs release various cytokines, including IL-6, but it is not always clear what are the main eliciting factors. We therefore hypothesize that the P2X7R could be important for IL-6 release. We measured the amount of IL-6 released from hPSC and mPSC after P2X7R activation/inhibition (Figure 4). In hPSC, IL-6 release was significantly reduced with AZ10606120 treatment (Figure 4a), indicating that already in the basal conditions the P2R7R was activated, similar to what was seen in the cell proliferation (Figure 2a). BzATP stimulation also showed a similar biphasic trend on IL-6 release as on cell proliferation. Mouse PSCs, isolated from either P2X7R ^wt^ or P2X7R ^P451L^ mice, showed a significant and robust IL-6 release after stimulation with 100 μM BzATP and some already with 10 μM BzATP (Figure 4b,c). In PSCs from both mice strains, the addition of AZ10606120 inhibited the stimulatory effect of 100 µM BzATP. However, AZ10606120 made the effect of 10 µM BzATP stimulatory. The millimolar concentration of BzATP was very effective in decreasing IL-6 release in both mouse models and, interestingly again, the pore inhibitor A438079 rescued this inhibitory effect and increased the IL-6 secretion. The anomalous rescue of inhibitors seemed to be similar in both mPSCs from P2X7R ^wt^ and P2X7R ^P451L^ mice. Together, these data show that the P2X7R is involved in IL-6 release from PSCs in both humans and mice.

### 3.5. Activation of P2X7R in PSCs Stimulates Migration of Cancer Cells

**Figure 5:**
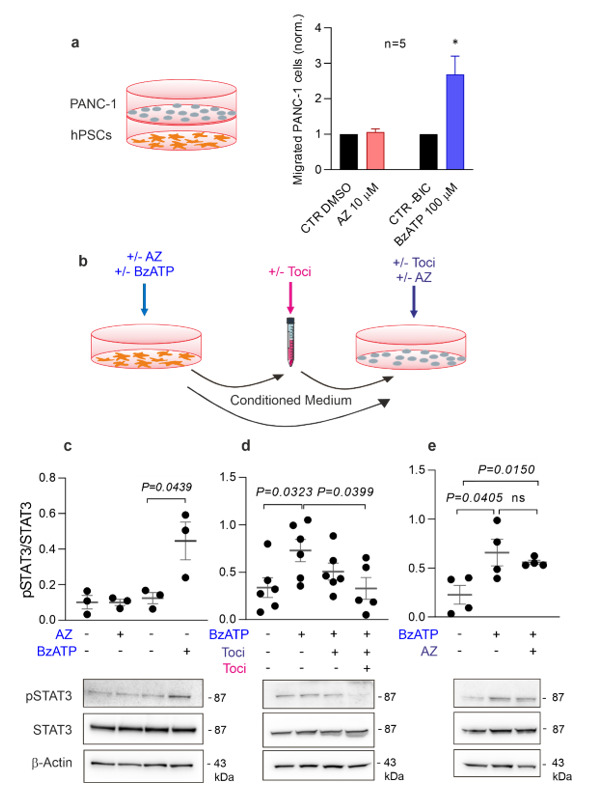
Effect of hPSCs and conditioned media on PANC-1. (**a**) Schematic diagram of Boyden chamber experimental setup showing PANC-1 cells in the upper chamber and hPSCs in the lower chamber. The histogram shows the number of migrated PANC-1 cells after co-incubation with hPSCs treated with either AZ10606120, AZ, (10 μM) or BzATP (100 μM) normalized to either a DMSO or –BIC control, in which the drugs were dissolved, respectively. * *p* < 0.05. (**b**) Schematic diagram of the experimental setup for (**c**,**e**), where conditioned media was harvested from hPSCs and added to PANC-1 cells. The hPSCs were treated with +/− P2X7R agonist (BzATP, 100 μM) or antagonist (AZ, 10 μM) (blue), the conditioned media was treated with +/− 10 ng/mL Tocilizumab (Toci) (pink), and PANC-1 cells were treated with either +/− 10 ng/mL Toci and +/− 10 μM AZ (purple). (**c**,**e**) Representative Western blots and quantification of STAT3 activation in PANC-1 cells after stimulation with conditioned media from hPSCs. STAT3 activation is reported as pSTAT3/total-STAT3 (**c**). The same experiment has been repeated inhibiting IL-6 receptors on PAN-C1 cells or on PANC-1 cells and CM (**d**). PANC1 pre-treatment with AZ10606120 (10 μM) has been performed to exclude a potential impact of P2X7R activation on STAT3 activation in PANC1 (**e**). β-actin was used as a loading control. Significant P values are shown in the graph and ns indicates non-significance.

Next, we tested the hypothesis that activation of the P2X7R in PSCs could induce cancer cell migration. Hence, we performed a co-culture migration assay in a Boyden chamber, where human pancreatic cancer cells, PANC-1 (upper chamber), were cultured in the presence of hPSCs (lower chamber) treated with P2X7R agonist or inhibitor (Figure 5a). Aphidicolin was added in both chambers to stop proliferation that could interfere with the assay and serum was also kept low (1%) in both chambers to avoid its direct role as a chemoattractant. The number of migrated PANC-1 cells was significantly increased when hPSCs were treated with BzATP (100 μM) (Figure 5a). Treatment with AZ10606120 did not affect PANC-1 migration, though some inhibitory effect on IL-6 release was observed (Figure 4a). To test that the drugs did not directly affect PANC-1 migration, we set up a similar assay without PSCs and observed that PANC-1 cells did not show significant migration (Supplementary Materials Figure S3). These data indicate that P2X7R activation in PSCs leads to the release of chemoattractants, possibly IL-6, into the media, which can affect the function of nearby cancer cells.

### 3.6. IL-6R Neutralization with Tocilizumab Prevents Activation of the STAT3 Pathway in Pancreatic Cancer Cells Induced by Conditioned Media from hPSCs

Next, we tested the hypothesis that chemoattractants released from the PSCs stimulate cancer cells by activating the STAT3 pathway. First, we collected the conditioned media from hPSCs treated with agonist/inhibitor of the P2X7R and used this to stimulate PANC-1 cancer cells for 30 min (Figure 5b). The PANC-1 proteins were extracted and the active form p-STAT3 and total STAT3 were quantified with Western blot analysis (Figure 5c–e). The pSTAT3 levels were significantly increased in PANC-1 exposed to the conditioned media from the hPSCs treated with BzATP (100 μM), whereas it was not affected with the receptor inhibitor (Figure 5c). The pSTAT3 increase occurred early at 30 min incubation. Thus, hPSCs secrete factors that stimulate STAT3 activation in pancreatic cancer cells.

To test our hypothesis that IL-6 is responsible for the activation of the STAT3 pathway in pancreatic cancer cells, we performed experiments where we neutralized the IL-6 receptors (IL-6Rs) using the IL-6R neutralizing monoclonal antibody Tocilizumab (Figure 5b). IL-6Rs can be found in two different forms: one integrated in the cell membrane and one soluble. Therefore, we designed two protocols. First, we added conditioned media from BzATP-stimulated hPSCs to PANC-1 cells, which were pretreated directly with Tocilizumab (10 ng/mL) (Figure 5b,d). This procedure would only affect the membrane form of IL-6Rs directly, and there seemed to be some reduction in STAT3 activation, though no significance reached (Figure 5d). Second, we added Tocilizumab to the PANC-1 cells as well as to the conditioned media to neutralize the soluble IL-6Rs (Figure 5b,d). This resulted in a significant reduction of pSTAT3 levels. Finally, since PANC-1 cells also express the P2X7R [22] and with the likelihood that some BzATP remained in the conditioned media, we wanted to confirm that the STAT3 activation happened through the P2X7R activation in the PSCs, and not by direct activation of the P2X7R in the PANC-1 cells. Therefore, PANC-1 cells were pretreated for 30 min with the P2X7R inhibitor AZ10606120 (Figure 5e). Pretreated cells did not show any significant reduction in pSTAT3, indicating that STAT3 activation requires IL-6 stimulation rather than P2X7R activation in pancreatic cancer cells.

### 3.7. hPSCs and PANC-1 Cells Release ATP

**Figure 6:**
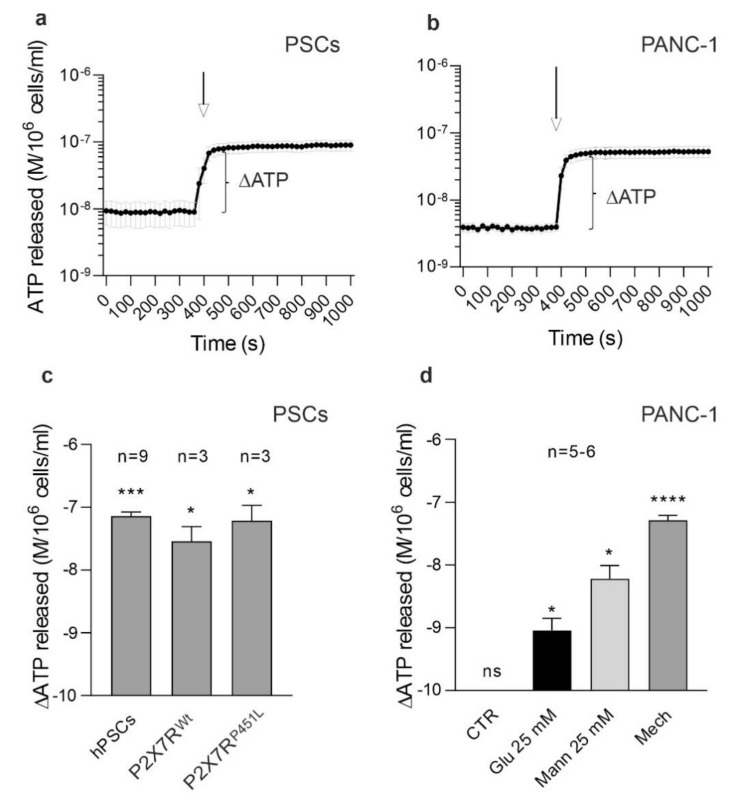
ATP release in PSCs and PANC-1. (**a**,**b**) Time course of ATP release in PSCs and PANC-1 cells after mechanical stimulation by injection pump showing the basal and stimulated ATP release (see methods). (**c**) The ATP release in response to the mechanical stimulus in the three types of PSCs. ATP quantification is expressed as ΔATP (M/10^6^ cells/ml, log axis) from basal to stimulated condition. (**d**) Effect of increasing substrate glucose from 5 to 25 mM (Glu 25 mM) or cell volume effects due to mannitol (Mann 25 mM) (manual addition) or mechanical stimulation (Mech) (pump injection). (**c**,**d**) Data were log transformed and maximal ATP release compared to the basal ATP, as depicted in (**a**,**b**), was tested in paired *t*-test analysis for each condition and significance is indicated as * *p* < 0.05, *** *p* < 0.001, **** *p* < 0.0001, ns non-significant.

The last question we sought to answer was the origin of the extracellular ATP that stimulates the P2X7R. In the TME, several conditions could lead to ATP release from both PSCs and cancer cells. Most cell types release ATP in response to mechanical stress (shear stress, cell volume changes, etc.), and one might expect that mechanical stress would be present in a pancreatic tumor, where significant pressures have been detected [39]. Therefore, we stimulated human and murine PSCs and PANC-1 cells with mechanical stimulation, induced by injection of a physiological buffer, and recorded ATP release in real time (Figure 6a–c). Our data show that all cell types tested responded with a fast ATP release. In addition, we tested PANC-1 cells after an osmotic stimulation with mannitol (25 mM), and metabolic stimulation with an increase in the glucose concentration from 5 to 25 mM (Figure 6d). Both types of stimuli also induced significant ATP release. These data show that both pancreatic cancer cells and PSCs release ATP into their environment, providing an agonist pool for the activation of the P2X7R.

## 4. Discussion

In the present study, we show that both human and mouse PSCs express the P2X7R and that this receptor evokes calcium signaling and stimulates collagen secretion. More importantly, the activation of the P2X7R shows a dual role in cell proliferation and death, and also elicits secretions of the cytokine IL-6. In turn, IL-6 released from PSCs stimulates STAT3 activation in pancreatic cancer cells, indicating that the P2X7R is an important factor in the PSC-cancer cell crosstalk.

We employed RLT-PSC as a suitable model for hPSCs in our study because these cells express several PSCs markers, such as desmin, vimentin, GFAP and α-SMA, as well as the P2X7R, which is functionally similar to the receptor in mPSC. Our data show that P2X7R activation promotes cell proliferation in both hPSCs and mPSCs. In the hPSCs, the receptor seems to have a high basal activity that stimulates cell proliferation, as this could be inhibited with the inhibitor AZ10606120, and low concentrations (10–100 μM) of the BzATP agonist showed negligible effects. Similar observations have been made on pancreatic cancer cell lines, beta-cell line, glial cells, and HEK293 expressing the P2X7R [22,37,40,41]. High basal activity of the receptor could be attributed to the high metabolic activity and significant release of ATP with metabolic substrates available in the media (Figure 6). It is reported that trophic effects in human cells are due to the P2X7B splice variant and/or the P2X7A-P2X7B heterotrimer [42]. In primary mPSCs, which proliferate slower in basal condition, the addition of low concentrations (10–100 μM) of the agonist BzATP had clear pro-proliferative effects. In mPSCs, both splice variant A and K are expressed and support proliferation [34], and variant K is activated by lower agonist concentrations and not affected by the P415L mutation [43]. In both cell types (hPSCs and mPSCs), high concentrations of BzATP (1000 μM) led to a lower cell proliferation and an increased cell death rate (Figure 2 and Figure 3), usually ascribed to the pore-formation [22,38,44]. The P2X7R ^P451L^ mutation affects ion channel/pore formation [45,46,47], and we observed lower calcium influx, though we did not observe marked differences in the downstream effects on BzATP-induced proliferation or IL-6 release (see below) in isolated mPSCs. Several explanations can be offered. Perhaps the remaining ion channel/receptor function in the P4521 mutant is sufficient to drive signaling to cell proliferation and cytokine release in mPSCs. Alternatively, it is possible that the K variant is more important in the proliferation of mPSCs. Furthermore, we cannot exclude that BzATP can at least partially activate other receptors, such as P2X4, though their effect on proliferation can be quite different to that found in our cells [48,49].

Nevertheless, we also used two different P2X7R inhibitors and some interesting observations emerged. Treatment with the P2X7R negative allosteric modulator AZ10606120 inhibited proliferation in all PSC types in basal state and with 10–1000 µM BzATP. The second competitive inhibitor, A438079, did not decrease the basal proliferation rate in all PSCs studied here. The inhibitor seemed to rescue, at least partially, from the high BzATP effect in hPSC. Interestingly, in our earlier studies, the inhibitor blocked the pore function and increased proliferation of mPSCs and PDAC cells, though cells were stimulated with 1–5 mM ATP rather than 1 mM BzATP [22,34]. The difference could be due to higher affinity of BzATP versus ATP, different agonist/antagonist competition and/or effects on other P2 receptors. Interestingly, A438079 decreased the pro-proliferative effect of 100 μM BzATP in mPSCs from P2X7R^wt^ mice; in contrast, in PSCs from P2X7R ^P451L^ mice it did not. It seems that the reduced channel/pore function in the P415L mutants can increase the number of mPSCs; in fact, we noticed a higher number of PSCs isolated from pancreas of P2X7R ^P451L^ mice compared to P2X7R ^wt^ mice. In contrast, the number of PSCs isolated from the Pfizer P2X7R knockout mice was lower than in the wild-type mice (both on the C57BL/6J background) [34].

In conjunction with our earlier study on PSC from P2X7R knockout mice [34], our results indicate that the P2X7R is important in the regulation of cell viability and proliferation; therefore, the number of PSCs in the pancreas. PSCs are important fibrogenic cells and in a previous study on an orthotopic pancreas cancer model we have noted that AZ10606120 treatment markedly reduced tumor fibrosis and collagen deposition [23]. In the present study, we confirm that the P2X7R stimulates collagen I secretion in vitro (Figure 3c,d). The PSCs have many similar features to hepatic stellate cells and the P2X7R may be an important target in combating pancreatic fibrosis and injury, similar to what has been seen with hepatic stellate cells in the liver [50,51].

The P2X7R has shown itself as a multi-faceted receptor, and the most important finding in our study is that the stimulation of this receptor also induces IL-6 secretion in both hPSCs and mPSCs. The effects of BzATP concentrations on IL-6 release parallel those on cell proliferation (Figure 2 and Figure 4), though there were some disparities between the effects of inhibitors in cell proliferation and IL-6 release. Interestingly, IL-6 secretion was enhanced when the effect of an inhibitory high dose of BzATP (1000 μM) was curbed with A438079. Additionally, it seems that in this condition mPSCs from P2X7R ^P451L^ mice secreted more IL-6 than cells from P2X7R^wt^ mice, but significant difference was not reached on the available data. Classical cytokines released by the P2X7R stimulation are inflammasome/NLRP3-associated IL-1β and IL-18 [24]. However, it has been reported that P2X7R stimulation causes NLRP3-independent IL-6 release in fibroblasts, neurons, astrocytes, microglia, and retinal cells [52,53,54,55,56], e.g., via mechanical stress, ROS signaling and exocytosis. However, IL-1β can upregulate transcription and release of IL-6, and thus, potentiate inflammation [57,58]. The P2X7R also stimulates proliferative and survival signaling involving ERK1/2 signaling [59] and PI3/Akt and NFκB [60]. These signaling pathways and multiple cytokines are important in the activation of PSCs and PDAC development and progression [61,62], but detailed mechanisms operating between P2X7R activation and IL-6 release in PSCs remain to be explored.

Previous studies have shown that “basal” IL-6 released from PSCs plays a leading role in activating the JAK/STAT3 pathway in pancreatic cancer cells [7,13,14,16]. Since extracellular ATP might be high in the TME of PDAC [4], our question was whether the P2X7R activation may impact or initiate this pathway. A co-culture of hPSCs and PANC-1 cells showed that the P2X7R stimulation of hPSCs leads to the secretion of a chemoattractant that promoted PANC-1 migration (Figure 5a). This is in agreement with the previous study on mPSC [23]. In the present study, we show that the chemoattractant and activator of cancer cells is IL-6 (Figure 5). First, conditioned media stimulated STAT3 activation in cancer cells. Second, the monoclonal antibody Tocilizumab, already used in clinic to inhibit IL-6R, prevented STAT3 activation in cancer cells. Notably, IL-6Rs are present in two different forms: in the cell membrane and as soluble molecules. In the first case, IL-6 binds to the membrane receptor and the activation of the pathway is mediated by the membrane-bound β-receptor glycoprotein 130 (gp130); in the second case, IL-6 binds the soluble receptor, and the complex migrates to the membrane, where gp130 is uniformly expressed, activating the pathway [63]. In fact, in our setup, STAT3 activation resulted in a modulated response depending on whether the membrane IL-6R (on PANC-1) or both the membrane and the soluble (conditioned media) forms of IL-6R were inhibited with Tocilizumab (Figure 5).

Preventing STAT3 activation could be an important therapeutic approach as STAT3 is one of the most known transcriptional factors associated with tumor progression. In PDAC, STAT3 activation promotes cell proliferation, migration, and invasion, as well as tumor stemness [64,65]. A recent study shows that increasing levels of pSTAT3 are present both in the TME and cancer cells, and they have different roles in tumor progression [66]. While STAT3 activation in the TME is important in the first stages of tumor development, STAT3 activation in cancer cells is more associated with the metastatic process. Furthermore, a highly activated STAT3 pathway is associated with chemoresistance and overall poorer prognosis in PDAC and gastric cancer patients [65,66,67].

So far, we discussed the evidence that P2X7R stimulation of PSCs causes the release of IL-6, which stimulates STAT3 signaling in pancreatic cancer cells. However, where does the extracellular ATP come from? There are many sources of eATP that could be relevant in the physiology and pathophysiology of the pancreas. Acini and ducts release ATP with agonists, mechanically, via pH, bile acids, etc. [20,68,69]. In the present study, we show an ATP release from mechanically perturbed PSCs and cancer cells, which is quite realistic as PDAC is a solid tumor showing high interstitial pressure [39,70,71]. Cancer cells have higher substrate supply/metabolism [72,73], and, as we show, they have increased ATP release with increased glucose in the media (Figure 6). Thus, our data indicate that in the TME of PDAC we might expect higher concentrations of eATP, as shown recently [4,21]. Accurate quantification of eATP concentrations in TME will require novel dynamic methods circumventing high ectonucleotidase activities.

In conclusion, in a TME rich in eATP, P2X7R activation in PSCs promotes IL-6 release that through STAT3 activation in cancer cells would promote tumor progression. This novel signaling pathway in between fibrogenic PSCs and cancer cells in the TME of PDAC could present a possible therapeutic approach to prevent fibrosis and STAT3 activation through P2X7R inhibition as well as IL-6R neutralization by Tocilizumab.

## Figures and Tables

**Table 1:** RT-PCR primers used in this study.

Species	Target	Primer Sequence (5′-3′)	Size (bp)
Mouse	P2X7R A long	Fw: GGCACCGTCAAGTGGGTC	1594
		Rev: AGCGCCAGGTGGCATAGC	
	P2X7R short	Fw: TGCTTTCTGCAGGTCGGGGGT	221
		Rev: TCTGGGGTCTTGGAACTTCTTGGCC	
Human	P2X7R A (I)	Fw: CGGTTGTGTCCCGAGTATCC	284
		Rev: CCTGGCAGGATGTTTCTCGT	
	P2X7R A (II)	Fw: TATGAGACGAACAAAGTCACTCG	95
		Rev: GCAAAGCAAACGTAGGAAAAGAT	

**Table 2:** Antibodies for IF.

Primary Antibody	Catalog #	RRID	Dilution
α-SMA	Ab5694 (Abcam)	AB_2223021	1:400
Collagen I	Ab34710 (Abcam)	AB_731684	1:200
Desmin	Ab32362 (Abcam)	AB_731901	1:150
GFAP	Ab7260 (Abcam)	AB_305808	1:200
Vimentin	Ab8978 (Abcam)	AB_306907	1:200
P2X7 (C-terminal)	Ab109246 (Abcam)	AB_10858498	1:100
P2X7 (extracellular)	APR-008 (Alomone)	AB_2040065	1:100
**Secondary antibody**			
Goat anti-rabbit Alexa 488	A11008 (Thermo Fisher)	AB_143165	1:200
Goat anti-mouse Alexa 488	A11001 (Thermo Fisher)	AB_2534069	1:200

## Data Availability

The authors confirm that the data supporting the findings of this study are available within the article and its Supplementary Materials. Additional raw data supporting the findings of this study are available from the corresponding author (I.N.) on request.

## Supplementary Materials

The following are available online at https://www.mdpi.com/article/10.3390/cells10081928/s1, Figure S1: Role of P2X7R on cell proliferation in 0% FBS, Figure S2: Cell viability, Figure S3: PANC-1 migration.
