# Supplementary material for: The P2X7 Receptor Stimulates IL-6 Release from Pancreatic Stellate Cells and Tocilizumab Prevents Activation of STAT3 in Pancreatic Cancer Cells
Source: Cells. 2021 Jul 29;10(8):1928. doi: 10.3390/cells10081928 (PMC8391419; doi:10.3390/cells10081928)
Supplement: Supplementary file 1 [file cells-10-01928-s001.zip › cells-1222494-supplementary.pdf]

Supplementary Material

The P2X7 receptor stimulates IL-6 release from pancreatic stellate cells and Tocilizumab prevents activation of STAT3 in pancreatic cancer cells

Lara Magni, Rayhana Bouazzi, Hugo Heredero Olmedilla, Patricia S. S. Petersen, Marco Tozzi and Ivana Novak*

Section for Cell Biology and Physiology, Department of Biology, University of Copenhagen, 2100 Copenhagen, Denmark
* Correspondence: inovak@bio.ku.dk, Tel.: +45-3532-0275

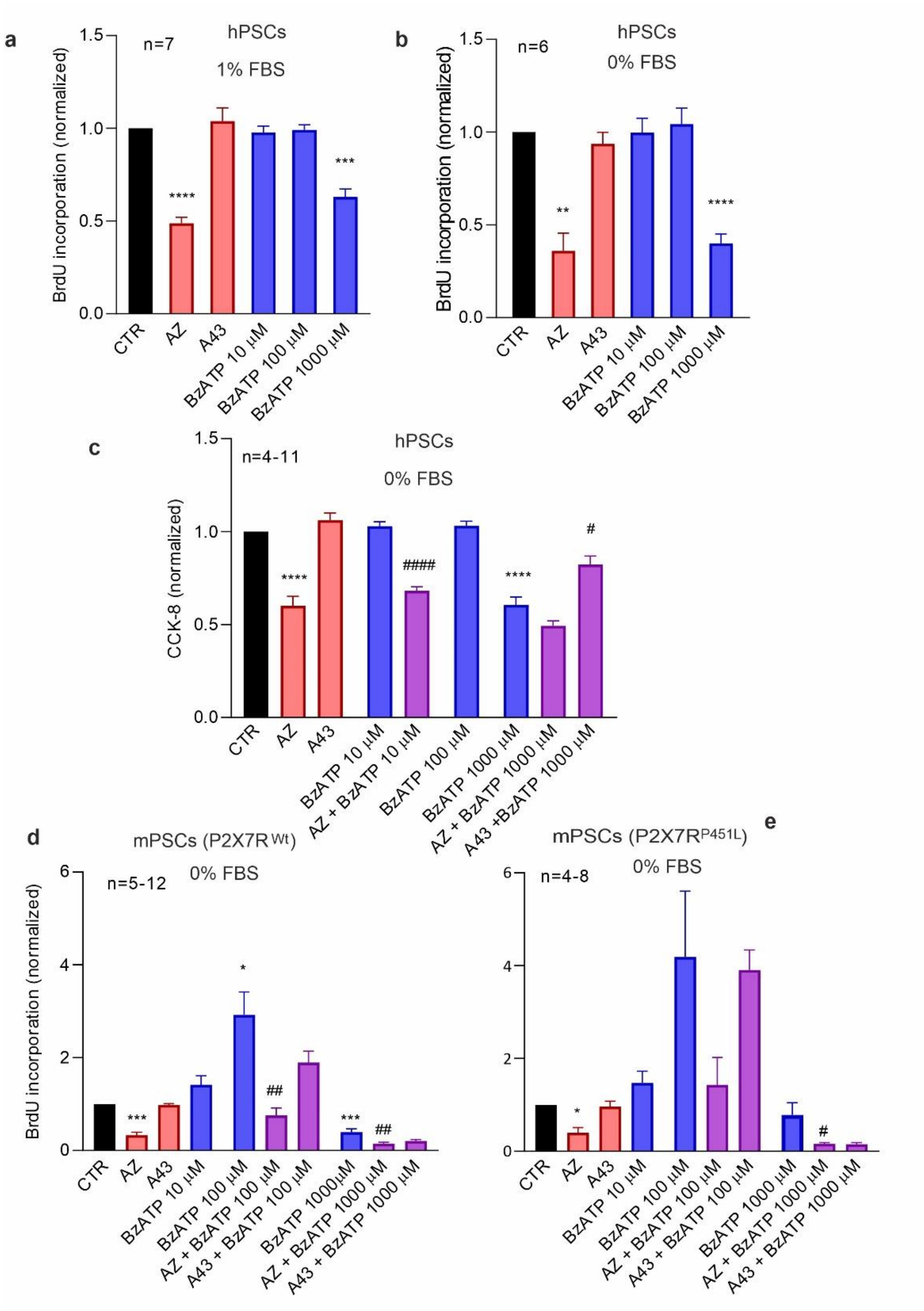

Figure S1. Role of P2X7R in cell proliferation. BrdU incorporation into hPSCs in (a) 1% FBS and (b) 0%FBS and mPSCs from (d) $P2X7R^{wt}$ and (e) $P2X7R^{P451L}$ mice cultured in 0% FBS. (c) CCK-8 absorbance in hPSCs 0%FBS. One-sample t-test followed by Bonferroni correction was performed and significance compared to the control is indicated as follow: * P <0.05, *** P <0.001, **** P<0.0001. Comparisons between agonist and agonist plus inhibitor have been evaluated with t-test and significance is indicated by # P < 0.05, ## P < 0.01, #### P <0.0001

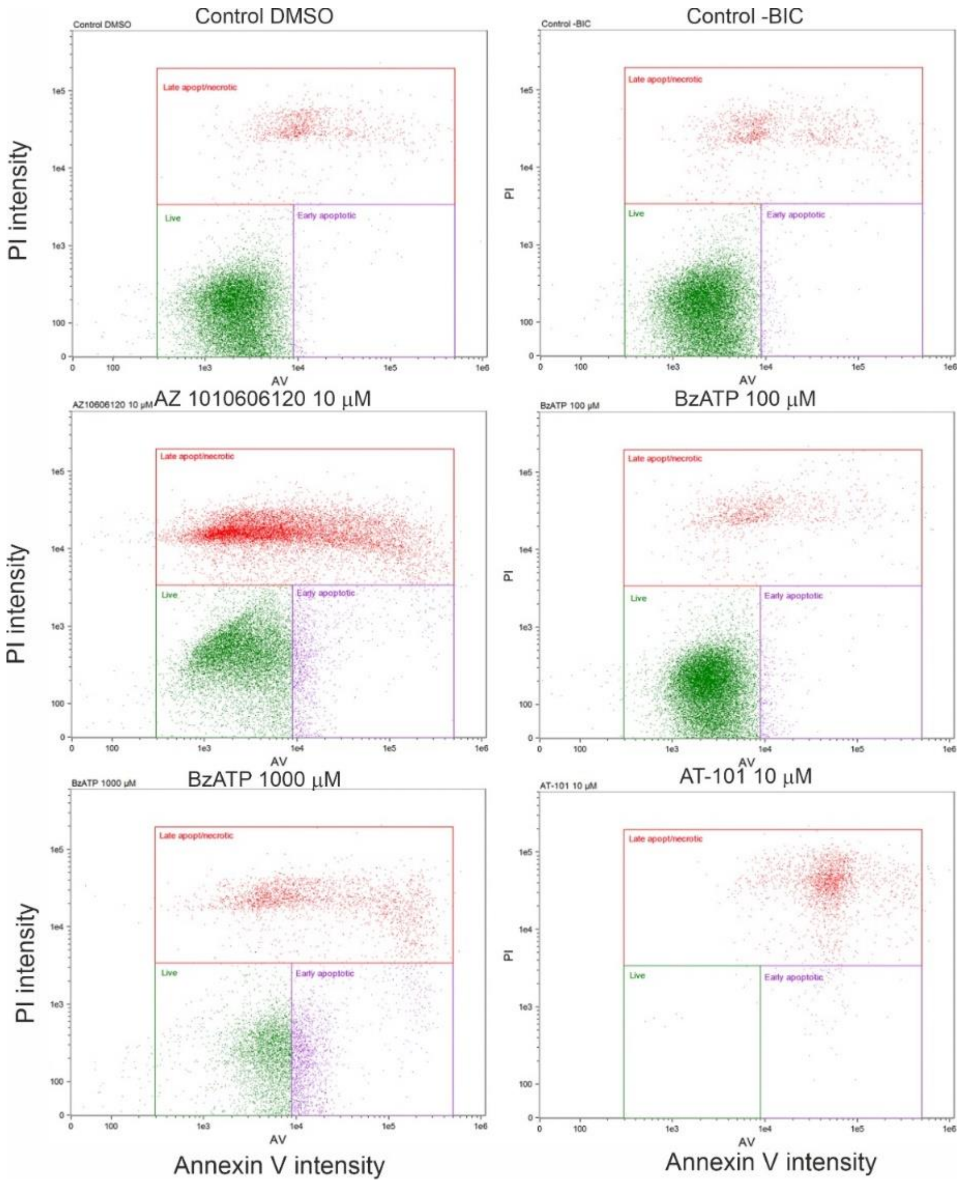

Figure S2. Cell viability. Representative picture from Flowsight analysis of hPSCs showing live cells (green), early apoptotic cells (purple) and cells in late apoptosis/ necrosis (red) after treatment with P2X7R inhibitor AZ10606120 (AZ) 10 µM or agonist BzATP (100-1000 µM). DMSO and –BIC were used as respective controls. AT101 10 µM was used as positive control.

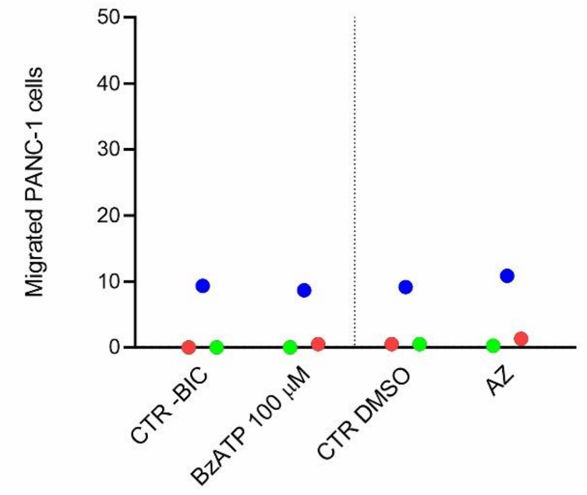

Figure S3. PANC-1 migration. Panc-1 cells were seeded in the upper chamber of a Boyden chamber. The lower chamber contained no hPSCs but agonist, antagonist and corresponding controls similar to tests depicted in Fig. 5a. The number of migrated cells in 3 different experiments is shown.
